# *Bartonella quintana* and *Rickettsia felis* in Gabon

**DOI:** 10.3201/eid1111.050861

**Published:** 2005-11

**Authors:** Jean-Marc Rolain, Olivier Bourry, Bernard Davoust, Didier Raoult

**Affiliations:** * Université de la Méditerranée, Marseille, France; † Centre International de Recherches Médicales, Franceville, Gabon; ‡ Direction Régionale du Service de Santé des Armées, Armées, France

**Keywords:** *Bartonella quintana*, fleas, monkey, primates, *Rickettsia felis*, Gabon, dispatch

## Abstract

We detected *Rickettsia felis* DNA in *Ctenocephalides felis* and *Bartonella quintana* DNA in 3 *Pulex irritans* fleas taken from a pet *Cercopithecus cephus* monkey in Gabon, sub-Saharan Africa. This is the first report of *B*. *quintana* in the human flea.

Bartonellae are gram-negative bacteria that cause several human diseases and are transmitted by various arthropods, such as lice, ticks, and fleas (*1*). *Bartonella quintana* is a worldwide fastidious bacterium that infects humans and belongs to the alpha subgroup of the *Proteobacteria*. Recent reports suggest that humans are the natural reservoir of *B*. *quintana* and that the human body louse is the vector (*1*). However, we have recently reported molecular detection of several *Bartonella* species, including *B*. *quintana*, in *Ctenocephalides felis* fleas from France, which suggests that fleas may be important vectors of human disease (*2*). Fleas are found worldwide on mammals and are vectors of several major zoonoses, including plague caused by *Yersinia pestis* (*3*), murine typhus caused by *Rickettsia typhi*, and fleaborne spotted fever caused by *R*. *felis* (*3*). More than 2,000 species of fleas exist worldwide. While some species are highly host specific, others are more catholic and will feed on numerous hosts, especially in the absence of their preferred host (*3*). Several flea species, including *Pulex irritans*, *C*. *canis*, *C*. *felis*, *Ceratophyllus gallinae*, *Ceratophyllus columbae*, and *Archaeopsylla erinacei*, may infest humans. In this study, we collected *P*. *irritans* (human fleas) and *C*. *felis* fleas on a pet monkey in Gabon and report for the first time the molecular detection of *B*. *quintana* in *P*. *irritans*.

## The Study

Four fleas collected from a pet monkey (*Cercopithecus cephus*) in Franceville, Gabon, were stored in 70% alcohol and sent to the World Health Organization (WHO) Collaborative Center for Rickettsial Reference and Research in Marseille, France, where molecular studies were performed in April 2005. Fleas were rinsed with distilled water for 10 min and dried on sterile filter paper in a laminar flow hood. Preliminary entomologic identification was performed by using reference taxonomic keys as previously reported (*4*).

Fleas were crushed individually in sterile Eppendorf tubes with the tip of a sterile pipette. DNA was then extracted by using the QIAamp Tissue Kit (Qiagen, Hilden, Germany) according to manufacturer's instructions. Rickettsial DNA was detected by polymerase chain reaction (PCR) with primers targeting the citrate synthase gene (*gltA*) as previously described (*4*). *R*. *montanensis* DNA was used as positive control, and negative controls consisted of laboratory uninfected flea DNA. *Bartonella* DNA was detected by PCR with 3 sets of primers targeting the intergenic spacer (ITS) gene, and the *B*. *quintana* spacers 336 and 894 as previously described (*4**,**5*). *B*. *elizabethae* DNA was used as positive control and uninfected fleas as negative controls. Additionally, fleas were identified at the species level after amplification and sequencing of a portion of the 18S rDNA gene as previously described (*4*). PCR products were purified, and DNA sequencing was carried out by using the d-Rhodamine Terminator cycle sequencing ready reaction kit with Amplitaq Polymerase FS (Perkin-Elmer, Coignieres, France) as described by the manufacturer. For all PCR products, sequences from both DNA strands were determined twice. Sequencing products were resolved by using an ABI 3100 automated sequencer (Perkin-Elmer). Sequence analysis was performed by using the software package ABI Prism DNA Sequencing Analysis Software version 3.0 (Perkin-Elmer). All obtained sequences were compared with those available in GenBank by using the nucleotide-nucleotide BLAST (blastn) program (http://www.ncbi.nlm.nih.gov/BLAST/).

Using morphologic taxonomic keys, 3 fleas were identified as *P*. *irritans* and 1 flea as *Ctenocephalides felis*. These findings were unambiguously confirmed when a 331-bp fragment of the 18S rDNA gene showed 99.7% and 99.4% homology with previous sequences of *C*. *felis* (GenBank accession no. AF423914) and *P*. *irritans* (GenBank accession no. AF423915). When *gltA* primers were used, *R*. *felis* (GenBank accession no. AF516333, 100% homology) was detected in the *C*. *felis*, whereas the *P*. *irritans* as well as negative controls were negative. Using the ITS primers for *Bartonella* spp., we detected PCR products in the 3 *P*. *irritans* fleas, whereas the *C*. *felis* flea and negative controls were negative. By sequencing the ITS gene–amplified fragments from these 3 fleas, we identified *B*. *quintana* (GenBank accession no. AF368396, 100% homology). Two PCR procedures targeting specific *B*. *quintana* spacers previously described (*5*) were carried out to confirm the results. By using these primers, *B*. *quintana* type 1 sequence was obtained for the spacer 336 (GenBank accession no. AY660705, 100% homology) and *B*. *quintana* type 2 sequence for the spacer 894 (GenBank accession no. AY660713, 100% homology). Thus, according to current guidelines for *B*. *quintana* typing (*5*), we have amplified genotype 2 of *B*. *quintana*.

## Conclusions

We present here the first molecular detection of *R*. *felis* in sub-Saharan Africa, Gabon (Figure). To date, 4 species of fleas have been associated worldwide with *R*. *felis* including *C*. *felis* (*3**,**6*), *C*. *canis* (*6*), *P*. *irritans* (*3*), and *Archeopsylla erinacei* (*4*). Thus, the amplification of *R*. *felis* in the *C*. *felis* flea from the monkey was not surprising but suggests that nonhuman primates may be infected as well as humans and may represent a reservoir of *R*. *felis*. The role of mammals, including rodents, hedgehogs, cats, dogs, and monkeys, in the life cycle and circulation of *R*. *felis* remains unclear and warrants further epidemiologic studies.

**Figure:**
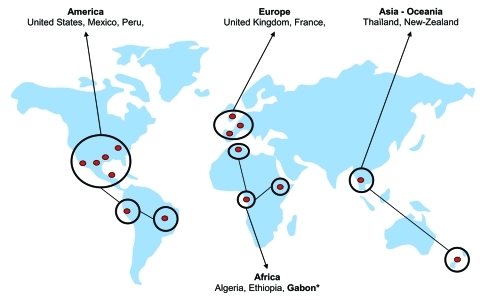
Detection of Rickettsia felis in fleas worldwide. *This study.

We report for the first time that the human flea *P*. *irritans* can be infected with *B*. *quintana*. Apart from the body louse, the natural vector of *B*. *quintana* in humans, we have previously detected *B*. *quintana* in *C*. *felis* fleas with a prevalence of 4.5% in a series of 309 fleas collected in various regions of France (*2*). Thus, our results confirm that *B*. *quintana* may be found in the human flea and may explain 2 clinical reports of chronic adenopathy attributed to *B*. *quintana* infection for which the only epidemiologic risk factor identified was the presence of fleas (*7**,**8*). Few reports of detection of other bartonellae in fleas have been made (Table). Recently, the rodent flea *Ctenophthalmus nobilis* has been found to be a competent vector of at least 2 *Bartonella* species, *B*. *grahamii*, which has previously been associated with human infection, and *B*. *taylorii* (*9*). In contrast, no evidence of either horizontal or vertical transmission was seen in bank voles (*Clethrionomys glareolus*) injected with *B*. *taylorii* maintained in an arthropod-free environment, which suggests that fleas may be essential for transmitting some *Bartonella* spp (*9*). In the study of Stevenson et al, *Bartonella* spp. were detected in 38 *Oropsylla hirsuta* and 3 *Oropsylla tuberculatus cynomuris* prairie dog fleas in United States (*10*). In addition, new *Bartonella* genotypes, whose medical importance is not yet known, were detected in *Pulex* fleas in Peru (*11*), in 5 *C*. *felis* collected from cats, and in a *Nosopsyllus fasciatus* collected from a *Rattus surifer* specimen in Thailand (*6*).

**Table:** Bartonella species detected in fleas worldwide

Species	Species of *Bartonella* detected	Country/animal (ref.)
*Ctenocephalides felis*	*B*. *clarridgeiae*	France/cat (*2**,**14*) Thailand/cat (*6*) New Zealand/cat (*15*)
	*B*. *koehlerae*	France/cat (*2*)
	*B*. *quintana*	France/cat (*2*)
*Ctenophthalmus nobilis*	*B*. *grahamii*	United Kingdom/bank vole (*9*)
	*B*. *taylorii*	United Kingdom/bank vole (*9*)
*Nosopsyllus fasciatus*	*Bartonella* spp.	Thailand/rodent (*6*)
*Oropsylla hirsuta*	*Bartonella* spp.	United States/prairie dog (*10*)
*O*. *tuberculatus cynomuris*	*Bartonella* spp.	United States/prairie dog (*10*)
*Pulex* spp.	*Bartonella* spp.	Peru/human (*11*)
*Pulex irritans*	*B*. *quintana*	Gabon/monkey (this study)

Although detection of *Bartonella* DNA is often reported from several sources, including fleas, mammals, and human samples, isolation of bartonellae by culture remains infrequent (*12*). Culture media and procedures used for *Bartonella* spp. have been highly variable and have questionable sensitivity (*12*). A novel chemically modified liquid medium that will support the growth of several *Bartonella* spp. has been recently developed and may provide an advantage over conventional blood agar culture for the isolation of *Bartonella* spp (*13*). The prevalence of *B*. *quintana* as well as other bartonellae in human fleas remains unknown, and this subject needs to be addressed to better define possible sources of *Bartonella* infections in humans.
